# Circulating Tumor Cell Persistence Associates with Long-Term Clinical Outcome to a Therapeutic Cancer Vaccine in Prostate Cancer

**DOI:** 10.3390/jpm11070605

**Published:** 2021-06-26

**Authors:** Ingrid Jenny Guldvik, Lina Ekseth, Amar U. Kishan, Andreas Stensvold, Else Marit Inderberg, Wolfgang Lilleby

**Affiliations:** 1 Department of Tumor Biology, Institute of Cancer Research, Oslo University Hospital, 0379 Oslo, Norway; 2 Institute of Clinical Medicine, University of Oslo, 0318 Oslo, Norway; 3 Faculty of Clinical Medicine, University of Stettin, 70-111 Szczecin, Poland; lina.ekseth@gmail.com; 4 Department of Radiotherapy, University of California, Los Angeles, CA 90095, USA; AUKishan@mednet.ucla.edu; 5 Department of Oncology, Østfold Hospital Trust, 1714 Kalnes, Norway; andreas.stensvold@so-hf.no; 6 Translational Research Unit, Department of Cellular Therapy, Oslo University Hospital, 0379 Oslo, Norway; Suso.Else.Marit.Inderberg@rr-research.no; 7 Department of Oncology, Oslo University Hospital, 0379 Oslo, Norway; WLL@ous-hf.no

**Keywords:** circulating tumor cells, prostate cancer, cancer vaccine, immune response, biomarker

## Abstract

De novo metastatic or recurrence of prostate cancer (PC) remains life-threatening. Circulating tumor cells (CTCs) are noninvasive biomarkers and provide unique information that could enable tailored treatment. This study evaluated the impact of CTCs in PC patients eligible for peptide vaccine therapy. Twenty-seven patients were tested for CTCs with the CellCollector^®^ device (Detector CANCER01(DC01)) during short-term androgen deprivation therapy (ADT) before cancer vaccine treatment (cohort 1) or salvage radiation (cohort 2). CTC counts were compared to clinicopathological parameters. In cohort 1, CTCs were correlated to immune responses, serum protein profiles, and clinical outcomes. In cohort 2, captured CTCs were further profiled for expression of PSMA, PAP, and PD-L1. Nine out of 22 patients (40.9%) in cohort 1 were CTC positive. These patients demonstrated vaccine-specific immune response (*p* = 0.009) and long-term prostate cancer-specific survival (log-rank, *p* = 0.008). All five patients in cohort 2 had CTCs at recurrence (count range 18–31), and 4/5 had CTCs positive for PSMA, PAP, and PD-L1. The DC01 CTC detection provides information beyond current clinical practice. Despite the small size of cohort 1, a correlation between CTC detection and outcome was shown.

## 1. Introduction

Prostate cancer is among the most common occurring malignancies globally, and despite the high effectiveness of definitive treatments in the primary setting, the disease will recur in 20–30% of patients [1]. Moreover, owing to the lack of screening programs for early detection of PC, emerging worrying statistics demonstrate that a larger proportion of patients present with more advanced PC and metastatic PC [2]. In Norway, PC is the second leading cause of cancer-related deaths after lung cancer. One man out of seven will develop PC during his lifetime, and more than 100,000 men die of prostate cancer in Europe each year. The probability of developing PC sharply increases in the sixth decade of life and further increases after age 70 [3]. The aging of the current population means that the disease will become an even more significant public health issue in the future.

Additional predictive biomarkers are urgently needed to improve the standard clinical decision model used in the routine staging of this disease (T stage, Gleason score, serum prostate-specific antigen (PSA), and bone scan) [4].

Several studies have confirmed the predictive and prognostic value of circulating tumor cell (CTC) detection as a monitoring method for treatment response in castration-resistant PC patients [5,6].

Recent reports have shown the efficacy of an in vivo capture device (CellCollector^®^, Detector CANCER01, DC01, Gilupi GmbH, Potsdam, Germany) in men with high-risk non-metastatic PC treated with definitive therapy [7]. This novel antibody-coated medical assay captures epithelial cell adhesion molecule (EpCAM) positive circulating cells that allow enumeration and further characterization of these cells.

**Figure 1:**
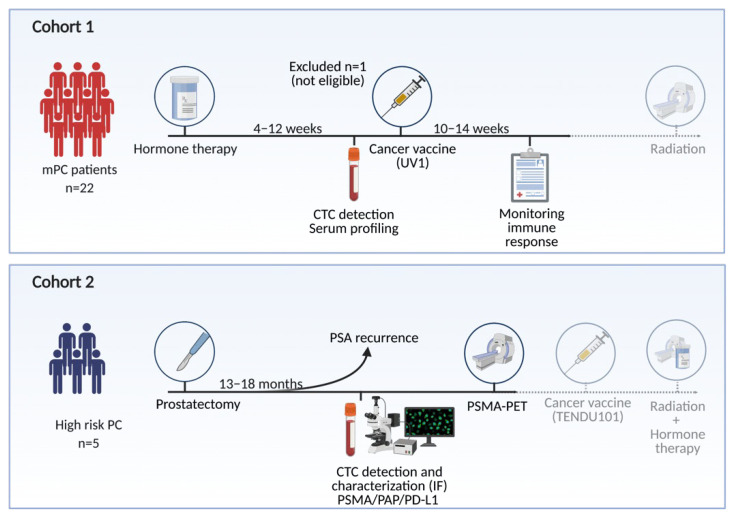
Overview of study. Two independent patient cohorts has been investigated for the presence of CTC. Figure created with Biorender.com.

We have applied the DC01 to detect CTCs in patients enrolled in two different studies with de novo metastatic PC (mPC, cohort 1) receiving ADT and a synthetic long peptide vaccine that targets telomerase (UV1^®^), and with biochemical relapse after radical prostatectomy (bPC, cohort 2) (Figure 1). Here, we report the prevalence of CTCs and evaluate the associations to immune responses, serum protein, and long-term clinical outcome (cohort 1) and explore molecular features of CTCs present in biochemically relapsed PC before salvage radiation (cohort 2).

## 2. Materials and Method

### 2.1. Patients

#### 2.1.1. Cohort 1 (mPC)

In the mPC cohort, twenty-two patients participated in a phase I study with a therapeutic cancer vaccine (UV1^®^, Ultimovacs, Oslo, Norway), a second-generation hTERT peptide-based cancer vaccine [8]. The primary objective of this study was to determine the maximum-tolerated dose and safety. CTC capture and enumeration were performed at enrollment and blood samples were biobanked for biomarker discovery. The study was approved by the institutional protocol board, the Ethical Committee (EudraCT 2012-002411-26), and the National Medical Agents Authority (NoMA), and the study was registered at clinicaltrials.gov (NCT01784913) [9]. The study was approved by the Ethics Committee of Health Region South-East (protocol code A 2013/112 of date 17.03.2013). Written consent was obtained from all participants.

#### 2.1.2. Cohort 2 (bPC)

This pilot study consisted of five men (bPC) referred to salvage radiotherapy after prostatectomy with high-risk features defined by the EAU guidelines [4]. The study was initiated to test the possible utility of the DC01 to detect CTCs in a planned first-in-man phase I study with a new therapeutic peptide vaccine (TENDU101^®^, EudraCT 2020-000918-15, NCT04701021). The study was approved by the Ethics Committee of Health Region South-East (protocol code D 2020/143561 of date 9 September 2020). Written consent was obtained from all participants.

### 2.2. Laboratory Analyses

#### 2.2.1. Capture of CTCs

The detection of CTCs was performed utilizing the novel in vivo device CellCollector^®^CANCER01 (DC01) (Gilupi GmbH, Potsdam, Germany) that captures and enables enumeration of EpCAM positive tumor cells in the circulation [7]. An intravenous catheter (20-gauge, BD-Venflon™, Stockholm, Sweden) was placed into a cubital vein, and the DC01 was inserted into the catheter, dwelling for 30 min in the bloodstream. After being applied in patients, the tip of the CellCollector^®^ (DC01) was washed three times in phosphate-buffered saline (PBS) (Gibco) including 1.6 mg/mL (final) ethylenediaminetetraacetic acid (EDTA) (Roth). Bound cells were fixed with Acetone (VWR) for 10 min at room temperature, dried, stored at −20 °C, and transferred on dry ice to Gilupi GmbH, Potsdam, Germany for further processing.

#### 2.2.2. Immunocytochemical Analysis and CTC Enumeration in Cohort 1

All procedures were performed by an experienced operator who was blinded to patient characteristics. In brief, the cells were blocked and permeabilized with 3% bovine serum albumin (BSA) (Roth) and 0.1% Triton X-100 (Fluka) in PBS for 30 min. Primary antibodies, including anti-pan-cytokeratin-Alexa 488 (CK4, 5, 6, 8, 10, 13, 18) (C-11) (Exbio), anti-CK19-Alexa 488 (A53-B/A2) (Exbio), anti-CK7- fluorescein isothiocyanate (LP5K) (FITC) (Millipore), anti-EpCAM- fluorescein isothiocyanate (FITC) (HEA125) (Acris), and anti-CD45- Alexa 647 (MEM-28, Exbio), were added for 30 min. The DC01 was then rinsed three times with 3 mL of PBS, and the nuclei were counterstained with Hoechst 33342 (Invitrogen). CTCs were identified using a Zeiss Axio imager fluorescent microscope with a 20× objective. Fluorescent images were recorded with a Zeiss MRm camera. A cell was considered to be a CTC if it was positively stained for cytokeratin and/or EpCAM, it was negative for CD45, and certain morphological criteria for tumor cells were met: the presence of a nucleus with a round or ellipsoid shape and a cell size ranging from 4 to 50 μm. Leukocytes were defined as nucleated (Hoechst-positive), CD45-positive, and cytokeratin and/or EpCAM-negative cells, and were not counted.

#### 2.2.3. Immunofluorescence Staining for PSMA, PD-L1, and PAP in Cohort 2

Immunocytology was combined with immunofluorescence (IF) staining for PSMA, PAP, and PD-L1. The following criteria defined tumor cells: intact morphology, diverse cells (large cell bodies, irregular cell shapes, several cells next to each other/cluster), cell diameter ≥ 4 μm, distinct and positive nuclei staining by Hoechst, and at least one positive marker (PAP or PD-L1 or PSMA). Nucleated cells with tumor cell-like morphology, but lacking IF staining were reported, but counted as negative.

Cells attached to the DC01 were permeabilized in 1× PBS/0.1% GIBCO™ for 10 min, washed three times in 1× PBS, and blocked with 1× PBS/3% BSA (Roth) for 30 min. Cells were further blocked with PBS/3% normal goat serum (Invitrogen) for 30 min. Immunolabeling was performed for 30 min at room temperature in 45 µL of PBS/3% BSA (Roth) containing primary antibodies (mouse IgG1-anti PSAP/PAP (clone PASE/4LJ, unconjugated, Invitrogen, dilution 1:25) and rabbit-anti-PD-L1 XP^®^ (clone E1L3N^®^, unconjugated, Cell Signaling Technology, dilution 1:300)). Afterwards, samples were washed with PBS (Life Technologies: Carlsbad, CA, USA) twice for 10 min at room temperature with agitation and subsequently incubated for 30 min at room temperature with secondary antibodies protected from light. Goat-anti-mouse IgG1, Alexa Flour^®^647 (Invitrogen, diluted 1:300), and goat anti-rabbit IgG (H + L), Alexa Flour^®^555 (Life Technologies, dilution 1:400), were also prepared in 45 µL of PBS/3% BSA. Following two wash steps in PBS for 10 min at room temperature with agitation, the samples were incubated for 30 min at room temperature protected from light with the conjugated antibody solution PSMA-Alexa Flour^®^488 (clone k1h7, Novus Biological, diluted 1:100) in PBS/3% BSA. After washing with PBS for 1 min at room temperature, cells were counterstained with Hoechst 33342 (Sigma Aldrich, final concentration: 1 µg/mL in PBS/3% BSA) for 5 min at room temperature, washed with PBS for 1 min at room temperature, and air-dried for 5 min each (all steps protected from light). Images of stained cells were acquired using a fluorescent microscope (Axio Imager Carl Zeiss AG, Jena, Germany) combined with a monochrome camera. Filter set (Carl Zeiss AG) numbers used for microscopic evaluation were 49 (Blue), 52 (Green), 43 (Orange), and 50 (Dark.RED).

#### 2.2.4. Detection of UV1^®^ Vaccine-Specific T-Cell Response in Cohort 1

Peripheral blood in acid citrate dextrose (ACD) tubes was taken from patients before UV1^®^-vaccination, two weeks after vaccination, and then monthly until week 26, then every three months. A detailed description of the procedures mentioned herein can be reviewed in Lilleby et al., 2017 [9]. Peripheral blood mononuclear cells (PBMCs) were isolated, frozen, and stored before further analysis. PBMCs were then thawed and pre-stimulated with the three UV1^®^ vaccine peptides, and the UV1^®^-specific T cell proliferative response was tested. Briefly, PBMCs were pre-stimulated with UV1^®^ peptide at 10 µM for 10–12 days, and cytokines (IL-2, IL-7) were added on day 3. On day 10–12, the T cells were then re-stimulated with irradiated, peptide-loaded autologous antigen-presenting cells (APCs), and T-cell proliferation was determined in 3H-Thymidine incorporation assays. The stimulation index (SI) was calculated by dividing the counts of wells with either the mix of UV1^®^-peptides or the three single peptides comprising the vaccine by the mean count of wells containing no peptide. An SI ≥ 3 was considered a positive, peptide-specific response.

#### 2.2.5. Targeted Serum Profiling in Cohort 1

Relative quantification of serum proteins known to be implicated in the interplay between the immune system and tumorigenic processes was performed by proximity extension assay (PEA) technology (Olink Bioscience Service Center Uppsala, Uppsala, Sweden) [10]. Briefly, one microliter serum drawn at study inclusion was profiled by the Immuno-Oncology panel (v.1). All sample handling and laboratory analyses were performed blinded. Data were normalized to minimize both intra- and inter-assay variation and presented as normalized protein expression values (NPX), an arbitrary unit on a log2 scale. NPX values of the different proteins within each patient were then analyzed to associate CTC findings and immune response.

### 2.3. Statistical Analysis

All statistical analyses were performed using SPSS (version 20) or R (version 3.3.1). All tests were two-sided, and a *p*-value < 0.05 was considered to be statistically significant. CTC counts were stratified as negative or positive, with positive meaning at least one cell to meet the criteria as CTC: In cohort 1, a CTC was defined as EpCAM+/panCK+/CD45-, whereas a CTC was defined as PSMA+/PD-L1+/PAP+ in cohort 2. In addition, all cells had to show normal morphology by Hoechst. Mann–Whitney U test (MWU) was used to assess differences in continuous variables between CTC positive and CTC negative patients. Chi-square test and Fisher’s exact test were used to evaluate associations between categorical coded variables. Spearman rank correlations were used to determine correlations between the number of CTCs detected, serum levels of proteins, and the number of peptides involved in immune reactivity towards the cancer vaccine. Kaplan–Meier survival analysis with prostate cancer specific survival (PCSS) and overall survival (OS) as endpoints was used to evaluate surviving proportions of patients stratified by CTC status. Log-rank test was used to test for statistical differences in surviving proportions. Univariate Cox proportional hazards (Cox PH) modelling was used to calculate crude hazard ratios (HRs) and evaluate the individual association of CTC with PCSS and OS.

## 3. Results

Two cohorts of patients were investigated (Figure 1). Patients’ characteristics are summarized in Table 1. The median age of cohort 1 was 67 years and 66 years in cohort 2.

### 3.1. CTC Presence Predicts Long-Term Survival Benefit of a Therapeutic Cancer Vaccine

#### 3.1.1. CTC Detection Predicts Broad Immune Response to Therapeutic Cancer Vaccine

**Figure 2:**
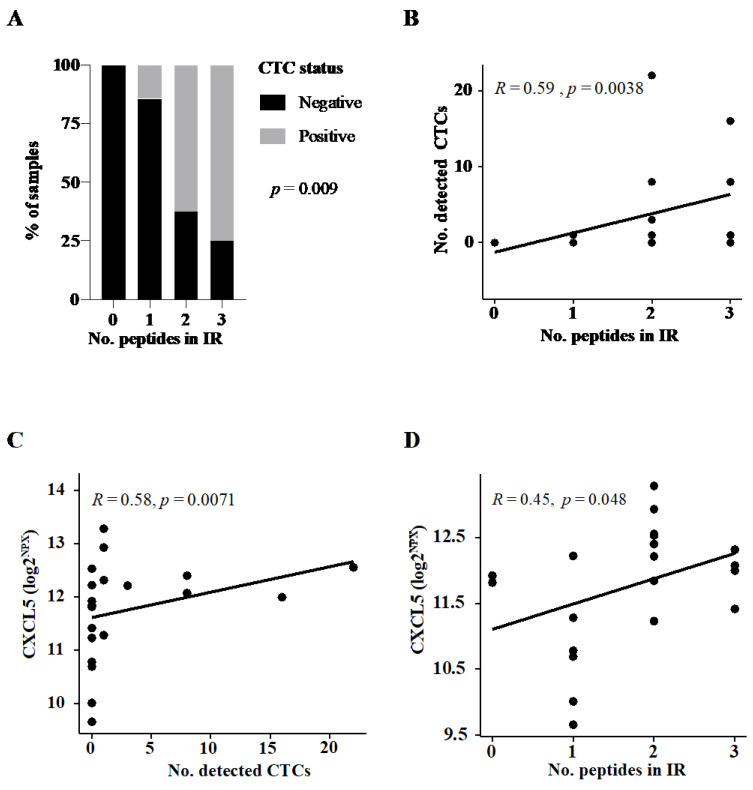
Assessment of association between circulating tumor cell (CTC) status, immune response, and serum proteins in cohort 1. (**A**) Percentage of CTC-positive patients according to the number of reactive UV1^®^vaccine peptides in immune response (IR). *p*-value reported on Chi-square test. Dot plot to assess the distribution of (**B**) the number detected CTCs according to the number of reactive peptides in immune response (IR) in vaccinated patients, (**C**) serum levels of CXCL5 across the number of CTCs detected, and (**D**) CXCL5 according to the number of reactive peptides in IR. Correlation coefficient and *p*-values are reported on the Spearman rank test (**B**–**D**).

In cohort 1, the median PSA was 3 ng/mL after starting with ADT (median duration on ADT 3.2 months, IQR 1.7–3.72) and 9 out of 22 patients (40.9%) had detectable CTCs. CTC positivity was associated with immunity towards two out of three UV1^®^ vaccine peptides (*p* = 0.009, Figure 2A). There was a direct correlation between the number of CTCs detected and the number of peptides involved in the immunity (Spearman rho 0.59, *p* = 0.004, Figure 2B).

In order to assess the circulatory immune microenvironment for CTCs, a targeted serum profiling by Olink technology was performed on samples collected in parallel of CTC capture. Both serum levels of CXCL5 and CD70 were significantly elevated in patients with CTC detected, whereas IL-18, ADGRG1, and HO1 were all downregulated (Table S1). Intriguingly, when assessing the relationship between a broad immune response (defined as reactivity to two or more peptides in the UV1^®^vaccine) and serum protein levels (Table S2), CXCL5 was also elevated in patients with a broad immune response. CXCL5 levels correlated positively both to the number of CTCs detected and to the number of peptides involved in the immune response (Figure 2C,D, respectively).

#### 3.1.2. Patients with CTC Show Survival Benefit of Therapeutic Cancer Vaccination

**Figure 3:**
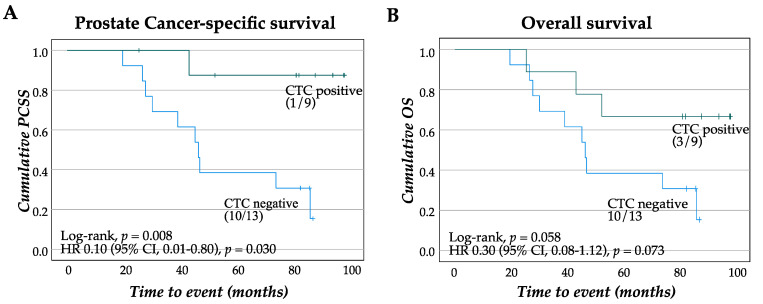
Survival estimates of patients stratified by CTC status. Kaplan–Meier plot and crude hazard ratio (HR) with confidence intervals (CIs) for PCSS (**A**) and OS (**B**) in cohort 1 (mPC) grouped according to CTC detection. PCSS: prostate cancer-specific survival, OS: overall survival.

Next, patients were categorized based on their CTC status and the long-term survival was assessed. Despite a small cohort, patients with CTC positivity illustrated long-term PCSS, with only 1 out of 9 succumbing to the disease at 80 months follow-up, whereas 10 out of 13 patients negative for CTC had PC-specific death (*p* = 0.008, Figure 3A). Overall survival was also improved for CTC-positive patients, with 3 out of 9 dead within the patient group positive for CTC and 10 out of 13 among the CTC negative patient group (*p* = 0.058, Figure 3B).

### 3.2. CTCs are Present at Biochemical Relapse and Express PSMA, PD-L1, and PAP

**Figure 4:**
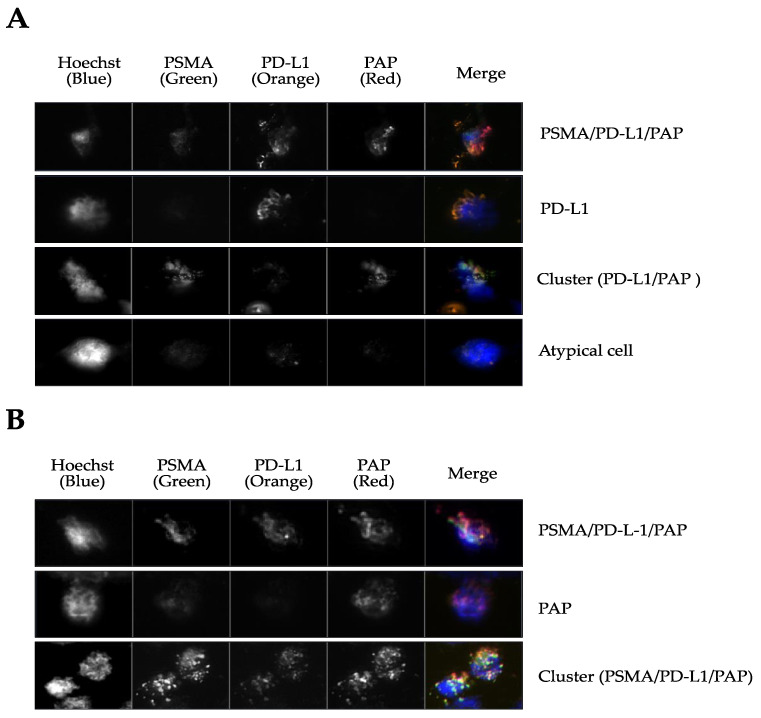
Immunofluorescence staining for PSMA, PD-L1, and PAP on captured CTCs in men with biochemical failure post-prostatectomy (cohort 2). (**A**) Examples of stained CTCs captured in patients with Gleason grade group 2; (**B**) examples of stained CTCs captured in Gleason grade group 5.

In a pilot study, CTC presence was assessed in five men referred to salvage radiotherapy after prostatectomy. All five patients presented with CTCs at biochemical relapse (range 18–31). Two out of the five patients had a negative PSMA-PET scan. Membrane staining of PSMA, PAP, and PD-L1 was assessed on the captured CTCs, as well as clusters (Table 2 and Figure 4). Four CTC samples were positive for all three markers, and patients with high Gleason grade group (4 and 5) had fewer CTCs with clusters (range 0–1), whereas patients with grade group 2 and 3 had CTCs with more clusters (range 3–4) (*p* < 0.0001). Further, the proportion of CTCs stained with all three markers increased with the Gleason grade (*p* = 0.005). In all samples, nucleated cells negative for PSMA, PD-L1, and PAP were found (Figure 4A).

## 4. Discussion

In the present study, we assessed the prevalence of CTCs in two clinically relevant settings: (a) patients with de novo metastatic PC and (b) patients with biochemical relapse PC referred to postoperative radiation. In both groups, we found considerable context-dependent evidence of CTCs even after ADT had been commenced (cohort 1) or when low serum PSA levels signaled tumor recurrence (cohort 2). Notably, targeting EpCAM is currently recognized as the only FDA-approved marker for detecting CTCs [11] and is recommended by the prostate cancer working group (PCWG3) guidelines [12]. Cancer cells of epithelial origin can retain stem cell-like features and constitute to further insight into the development of phenotypes and therapy failure [13,14]. The PCWG emphasizes the importance of clinical trials with a biomarker context. In the present study, CTC enumeration, immune response, and serum proteins were embedded in the disease state model.

Here, we could show that CTCs were detectable in 40.9% of patients with onset metastatic disease treated short-term with ADT and a detection rate of 100% in patients with biochemical relapse after prostatectomy. In both scenarios, patients had low serum PSA owing to ADT or resection of the prostate. The results give new insights into the biological behavior of PC, in patients with low serum PSA both due to ADT or prostatectomy.

It has been established that CTC is a prognostic marker in metastatic castration-resistant prostate cancer [6,9]. Surprisingly, patients positively stained for CTCs after the onset of ADT and before initiating the UV1^®^ vaccine had a survival benefit at median six-year FU post-vaccination. This is contrary to recent findings where a high count of CTCs signaled poor outcomes in those treated with life-long ADT [15]. We speculate that CTCs with epithelial features could be a source to antigens and act synergistically with the therapeutic peptide vaccine in stimulating cancer-specific immune cells, improving the outcome in some patients.

In support of this unexpected observation, we found that the number of CTCs detected in cohort 1 patients correlated to the broadness of the vaccine IR, demonstrated by the number of vaccine peptides involved [8]. Goldkorn et al. found that telomerase activity independently predicted overall survival in men with detectable CTCs [16]. The CTC presence could thus potentially boost the induction of an anti-telomerase IR by the UV1^®^vaccine. This opens up for further investigation on whether CTCs can be used to predict patients that will have a favorable response to arising immune therapies in prostate cancer [17].

The high prevalence of CTCs in cohort 1 during ADT raises some intriguing questions. It is well established that immunosenescence in older men leads to thymic involution and is related to the predominantly significant clinical detection of cancer [18]. However, ADT can reverse thymic involution, thereby recruiting naïve T-cells capable of forming lymphocyte infiltrates in the primary tumor [19,20]. Of note, the typical immune-pathological cell picture is governed by suppressed immunity in prostate cancer patients when treated with ADT [21]. However, ADT can lead to androgen receptor amplification and programmed cell death. Increased antigen presentation can assist in the often-seen sustained response in biochemical responding patients [22].

On the other hand, in those with a negative CTC finding (59.1% in cohort 1), ADT could reset cancer cells to senescence, shedding typical epithelial surface markers contributing to an immune mimicry. It has been recently described that secretory stimuli in the microenvironment of minimal residual disease can induce senescence [23]. Moreover, depending on the driver-mutation, the senescence-associated secretory phenotype of senescent tumor cells can have pro- and antitumorigenic effects [24]. Besides, cancer-associated fibroblasts producing CXCL5 are involved in promoting PD-L1 upregulation in tumor cells [25]. CXCL5 is often elevated in metastatic PC patients, increases with tumor apoptosis, and is thus considered as a relevant therapeutic target [26]. CXCL5 is involved in recruiting immune cells to the tumor, including myeloid-derived suppressor cells, contributing to tumor immunoresistance (reviewed in [27]). Our study found the chemokine CXCL5 to be associated with the number of CTCs and immune response. In line with previous reports, this could be the response of the cancer and its microenvironment to an immune attack, suggesting that CXCL5 is a potential Achilles’ heel. Combination treatment could be required to overcome such resistance mechanisms and to have a sustained and broad immune response.

In cohort 2, the number of CTCs detected was independent of PSA, and could also be detected at low PSA levels. CTCs survive only for a short time in the blood circulation [28]. Chen et al. showed that the finding of CTCs by the DC01 was reproducible at different time-points [29]. The in vivo DC01 device, previously tested in men after surgery, had a detection rate of 34% obtained three months after prostatectomy. In our study, a significant number of CTCs were detected more than one year after surgery in a prognostic high-risk group. Therefore, they likely originate from clinically significant minimal residual disease after primary radical resection of the prostate. In line with this, the CTC positivity in our study was supported by observing that CTC count was independent of PSA. Many recent basic science findings point toward the possible early genesis of a so-called immune tolerance [30]. This is in line with Benko et al., who found higher expression of EpCAM positivity in patients with high-grade Gleason score and T stage, and that EpCAM expression was a significant predictor of shorter biochemical recurrence-free survival [31]. Despite the normally long tumorigenesis of primary PC, CTCs may have accelerated clonal evolution, enabling them to spread.

Interestingly, CTCs from cohort 2 stained positively for PSMA with PD-L1 and PAP. The presence of CTCs was independent of PSA values or PSMA-PET findings. This is in line with the findings of Cieslikowski et al., who found presence of CTCs in patients with no evidence of metastasis by imaging [32]. PSMA is a transmembrane glycoprotein with catalytic properties, named glutamate carboxypeptidase II. It is not specific to prostate cancer, but has proven useful as it is highly overexpressed in prostate cancer cells in about 95% of the patients [33,34]. PSMA-PET has a sensitivity level that depends on the tumor volume. Sensitivity ranged from 42 to 98% and specificity from 71 to 99%. Thus, in patients with early biochemical relapse, not all will have sufficient minimal residual disease to be detected by PSMA-PET.

The expression of PD-L1 on CTCs has been linked to tumor immune evasion [10]. In the present study, Gleason grade and IF markers were correlated. The finding of a distinct phenotype in CTCs could provide a protective mechanism of CTC survival outside the tumor microenvironment.

Our study has limitations. Apart from the small sample size and the lack of baseline CTC measurement before initiation of ADT (cohort 1), CTCs are heterogeneous and might not at all express the epithelial marker. Using EpCAM as a positive selection marker may introduce a bias, but the prolonged in vivo detection time can lead to favorable enrichment of CTCs. Thus, by a pre-defined set of criteria, the probability of a false positive CTC decreased. Longitudinal observation of CTCs using the DC01 will be performed in the ongoing phase I TENDU101 study (NCT04701021).

In cohort 2, some nucleated cells captured by the DC01 were suspicious, but did not stain for either PSMA, PD-L1, or PAP and were disregarded as CTC. As these cells were not counterstained with CD45 or pan-cytokeratin, leukocyte origin cannot be excluded.

Although the survival data reported herein show great potential in the small study of the UV1^®^vaccine (cohort 1), we cannot exclude that immune responses triggered by other prostate antigens not covered by our assay contribute to improved clinical outcome in this cohort.

## 5. Conclusions

Our results indicate that implementation of the CTC detection could improve the shared decision-making process addressing targeted therapy for men with de novo and relapsed PC after prostatectomy. Notably, presence of CTCs during the onset of ADT and before the start of peptide vaccine was correlated to outcome. We found a substantial number of CTCs with the DC01 device, which could be a valuable clinical tool for assessing relapse, contextual treatment efficacy, and tailored therapy in men with PC.

## Figures and Tables

**Table 1:** Clinical characteristics of patients with metastatic prostate cancer (mPC) or bPC stratified by circulating tumor cell (CTC) status.

	Cohort 1 (mPC)			Cohort 2 (bPC)	
	CTC Status			CTC Status	
	***Positive***	***Negative***	***p=***	***Positive***	***Negative***
*n* (%)	9 (40.9)	13 (59.1)	<0.001	5 (100)	0 (0.00)
Time FU (month, median [IQR])	77.6 [52.6, 82.5]	46.6 [30.2, 76.9]			
Age (yr, median [IQR])	66.9 [59.2, 75.4]	66.8 [63.9, 73.9]	0.85	65.8 [57.4, 74.7]	
T stage (%)			0.72 *		
cT2	1 (11.1)	1 (7.7)			
cT3	6 (66.7)	7 (53.8)			
cT4	2 (22.2)	5 (38.5)			
pT2				2 (40.0)	
pT3				3 (60.0)	
Gleason grade group (%)			0.61 **		
2 + 3	0 (0.00)	0 (0.00)		3 (60.0)	
4	3 (33.3)	2 (14.3)		1 (20.0)	
5	6 (66.7)	11 (84.6)		1 (20.0)	
PSA values (ng/mL, median [IQR])					
PSA at diagnosis	26.0 [12.0, 72.0]	33.0 [12.0, 58.0]	0.95		
PSA after ADT	1.10 [0.40, 7.60]	3.00 [0.60, 9.20]	0.66		
PSA at relapse				0.26 [0.20,0.54]	
Time ADT/DC01 (mo., median [IQR])	3.42 [1.74, 3.72]	2.30 [1.64, 4.77]	0.92		
Time RP/DC01 (mo., median [IQR])				13.2 [7.02, 14.5]	
No. reactive peptides in IR (%)			0.009 *		
0	0 (0.0)	3 (23.1)			
1	1 (11.1)	6 (46.2)			
2	5 (55.6)	3 (23.1)			
3	3 (33.3)	1 (7.7)			

ADT: Androgen-deprivation therapy; IQR: interquartile range; FU: follow-up; IR: immune reaction; mo.: months; PSA: prostate-specific antigen; RP: radical prostatectomy; yr: years. * Chi-square test, ** Fisher’s exact test.

**Table 2:** Summary of immunofluorescence stained cells in individual patients.

						*p*= *
Patient ID	1	3	5	2	4	
Gleason Grade Group	2	3	3	4	5	
PSMA-PET status	+	-	+	-	+	
Total no. CTC	31	19	18	27	29	
PSMA, *n* (%)	0 (0.0)	0 (0.0)	5 (27.8)	0 (0.0)	0 (0.0)	0.96
PD-L1, *n* (%)	20 (64.5)	3 (15.8)	3 (16.7)	0 (0.0)	0 (0.0)	<0.001
PAP, *n* (%)	0 (0.0)	6 (31.6)	4 (22.2)	3 (11.1)	2 (6.9)	0.92
PSMA/PD-L1, *n* (%)	0 (0.0)	0 (0.0)	0 (0.0)	0 (0.0)	0 (0.0)	-
PSMA/PAP, *n* (%)	6 (19.4)	3 (15.8)	6 (33.3)	0 (0.0)	0 (0.0)	0.005
PD-L1/PAP, *n* (%)	0 (0.0)	0 (0.0)	0 (0.0)	0 (0.0)	0 (0.0)	-
PSMA/PD-L1/PAP, *n* (%)	5 (16.1)	7 (36.8)	0 (0.0)	24 (88.9)	27 (93.1)	0.005
Clusters (no.)	4 (12.3)	3 (15.8)	4 (22.2)	1 (3.7)	0 (0.0)	<0.0001
Nuclei+ cells (no. PSMA/PD-L1/PAP) †	4	5	8	11	0	-

* Chi-square test for trend; † not counted as positive CTC.

## Supplementary Materials

The following are available online at https://www.mdpi.com/article/10.3390/jpm11070605/s1, Table S1: Targeted serum profiling for differences between CTC status; Table S2: Targeted serum profiling for differences in immune response.
