# Supplementary material for: Circulating Tumor Cell Persistence Associates with Long-Term Clinical Outcome to a Therapeutic Cancer Vaccine in Prostate Cancer
Source: J Pers Med. 2021 Jun 26;11(7):605. doi: 10.3390/jpm11070605 (PMC8303369; doi:10.3390/jpm11070605)
Supplement: Supplementary file 1 [file jpm-11-00605-s001.zip › jpm-1241035-supplementary.pdf]

Table S1. Targeted serum profiling for differences between CTC status

Protein ($log2^{NPX}$, median [IQR])	CTC status		
	Negative	Positive	*p* =
	n=13	n=9	
CXCL5	11.41 [10.74, 11.88]	12.32 [12.07, 12.56]	0.007
IL18	9.17 [8.64, 9.48]	8.46 [8.41, 8.64]	0.009
ADGRG1	3.12 [2.03, 4.02]	1.72 [1.50, 2.04]	0.010
HO1	12.36 [12.09, 12.46]	11.87 [10.74, 12.19]	0.044
CD70	4.41 [4.06, 4.54]	4.70 [4.45, 4.80]	0.044
CRTAM	5.11 [5.06, 5.38]	5.02 [4.76, 5.10]	0.063
CXCL13	8.92 [8.88, 9.25]	8.74 [8.60, 8.86]	0.087
CD8A	9.46 [9.01, 10.07]	8.81 [8.63, 9.74]	0.102
GZMA	5.47 [5.20, 5.51]	5.20 [4.91, 5.26]	0.102
IL10	2.53 [2.35, 2.70]	2.39 [2.01, 2.50]	0.137
CXCL1	9.16 [8.90, 9.55]	9.72 [9.15, 9.86]	0.138
TNFRSF12A	6.42 [6.20, 6.61]	6.11 [6.00, 6.42]	0.138
CD40L	8.06 [7.52, 8.14]	7.03 [6.07, 7.83]	0.160
KLRD1	5.82 [5.58, 6.08]	5.62 [5.29, 5.86]	0.160
GAL9	7.75 [7.42, 7.91]	7.41 [7.35, 7.61]	0.184
CCL17	8.62 [8.18, 9.10]	9.01 [8.93, 9.22]	0.184
ICOSLG	5.73 [5.64, 6.61]	6.86 [5.79, 7.91]	0.184
TNFRSF9	5.80 [5.70, 5.95]	5.68 [5.43, 5.80]	0.210
FGF2	1.09 [0.75, 1.50]	0.82 [0.69, 0.86]	0.239
GZMH	4.98 [4.62, 5.24]	4.60 [4.41, 5.01]	0.239
VEGFC	2.91 [2.59, 3.29]	3.18 [3.07, 3.65]	0.239
CD27	8.19 [7.61, 8.26]	7.96 [7.91, 8.00]	0.239
NCR1	4.45 [4.16, 4.65]	4.22 [4.10, 4.32]	0.239
ADA	2.55 [2.19, 2.83]	2.34 [2.14, 2.49]	0.271
ANG2	4.82 [4.69, 4.99]	4.57 [4.49, 4.95]	0.271
CCL20	5.94 [5.46, 6.23]	5.61 [5.20, 5.74]	0.271
CX3CL1	6.77 [6.33, 7.03]	7.01 [6.63, 7.32]	0.305
CCL13	8.74 [8.18, 9.46]	9.21 [8.81, 9.45]	0.342
TWEAK	8.91 [8.71, 9.01]	8.79 [8.67, 8.97]	0.342
MMP12	6.92 [6.67, 7.25]	7.30 [6.88, 7.51]	0.342
VEGFA	8.97 [8.65, 9.18]	8.67 [8.55, 8.87]	0.342
CSF1	7.66 [7.52, 7.73]	7.53 [7.42, 7.71]	0.342
IL12RB1	1.91 [1.78, 2.09]	1.70 [1.63, 2.01]	0.382
CA9	3.84 [3.48, 4.11]	4.04 [3.85, 4.24]	0.425
EGF	10.13 [9.70, 10.85]	9.80 [8.73, 10.33]	0.470
IL6	3.98 [3.65, 4.54]	4.19 [3.92, 4.69]	0.470
PDCD1	3.36 [3.04, 3.83]	3.35 [3.03, 3.43]	0.470
CCL8	8.12 [7.58, 8.28]	7.92 [7.28, 8.15]	0.470
CCL4	8.02 [7.70, 8.42]	7.73 [7.55, 8.05]	0.470
MMP7	11.51 [11.17, 11.72]	11.25 [11.22, 11.43]	0.470
DCN	4.95 [4.84, 4.99]	4.93 [4.83, 5.45]	0.470
RSF4	3.49 [2.98, 3.56]	3.19 [3.08, 3.30]	0.470
PTN	2.07 [0.80, 2.85]	1.86 [1.25, 3.56]	0.514
CD244	6.30 [5.86, 6.48]	6.10 [5.92, 6.14]	0.518
GAL1	6.26 [6.12, 6.49]	6.20 [6.11, 6.34]	0.518
MICAB	4.04 [3.83, 4.28]	4.19 [3.86, 4.43]	0.518
NOS3	1.43 [1.09, 1.49]	1.25 [1.06, 1.47]	0.533
PIGF	8.49 [8.43, 8.70]	8.48 [8.34, 8.57]	0.569
TNFRSF21	7.88 [7.63, 7.99]	7.84 [7.58, 7.87]	0.569
CCL19	9.28 [8.62, 9.80]	8.70 [8.52, 9.58]	0.621
CCL3	5.81 [5.75, 6.13]	5.84 [5.52, 5.99]	0.621
CCL2	10.84 [10.57, 11.06]	10.88 [10.84, 11.08]	0.676
TGFB1	2.57 [2.27, 2.90]	2.37 [2.30, 2.64]	0.676
CD28	1.57 [1.43, 1.72]	1.54 [1.40, 1.61]	0.676
CD5	4.72 [4.53, 4.82]	4.79 [4.55, 5.01]	0.676
GZMB	3.71 [3.42, 4.07]	3.71 [3.19, 3.80]	0.676
IL8	6.61 [6.31, 6.81]	6.80 [6.39, 6.96]	0.732
CCL7	2.90 [2.48, 3.32]	2.71 [2.54, 3.00]	0.732
CXCL11	7.11 [6.29, 7.44]	6.46 [6.28, 7.01]	0.732
VEGRF2	7.40 [7.21, 7.60]	7.37 [7.28, 7.54]	0.732
FasL	6.02 [5.65, 6.29]	5.84 [5.73, 6.22]	0.732
CXCL10	8.49 [7.38, 9.18]	8.03 [7.82, 8.87]	0.732
IL12	6.49 [6.06, 6.75]	6.42 [6.33, 6.47]	0.732
CXCL9	7.72 [7.18, 8.48]	7.24 [7.02, 8.54]	0.790
TNFSF14	4.94 [4.63, 5.46]	4.89 [4.72, 5.50]	0.790
HGF	8.13 [8.06, 8.35]	8.21 [7.96, 8.43]	0.790
ANG1	10.28 [10.12, 10.37]	10.28 [10.20, 10.37]	0.849
IL7	6.00 [5.45, 6.42]	6.01 [5.42, 6.40]	0.849
TRAIL	8.21 [7.96, 8.26]	8.15 [7.96, 8.34]	0.849
CD40	10.80 [10.74, 11.08]	10.80 [10.53, 11.14]	0.849
PDGF_subunit_B	10.75 [10.68, 10.92]	10.82 [10.81, 10.83]	0.849
LAMP3	4.81 [4.50, 5.53]	5.09 [4.56, 5.32]	0.849
CASP8	6.91 [6.18, 7.63]	6.83 [6.30, 7.18]	0.849
CD83	2.57 [2.22, 2.65]	2.53 [2.28, 2.83]	0.849
TIE2	7.81 [7.69, 8.03]	7.86 [7.64, 8.03]	0.909
CD4	0.60 [0.45, 0.80]	0.59 [0.47, 0.80]	0.909
PDL1	4.79 [4.59, 5.07]	4.81 [4.58, 5.17]	0.909
CCL23	9.99 [9.85, 10.10]	10.03 [9.75, 10.16]	0.909
SDF1	1.24 [1.10, 1.30]	1.26 [1.03, 1.29]	0.909
PDL2	2.59 [2.48, 2.74]	2.57 [2.28, 4.50]	0.970
ARG1	3.56 [2.84, 4.51]	3.69 [2.79, 4.37]	1.000
IL4	1.41 [1.35, 1.46]	1.16 [0.98, 1.35]	1.000

IQR= Inter quartile range

Table S2. Targeted serum profiling for differences in immune response

**Protein ($log2^{NPX}$, median [IQR])**	**No. of peptides in IR**		
	**0/1** n=10	**2+** n=12	***p*=**
CXCL5	11.03 [10.52, 11.84]	12.26 [11.96, 12.54]	0.005
CCL17	8.36 [7.42, 8.66]	9.05 [8.93, 9.31]	0.009
CXCL1	8.90 [8.67, 9.29]	9.61 [9.15, 9.85]	0.017
CCL13	8.38 [7.94, 9.00]	9.26 [8.80, 9.64]	0.045
TIE2	7.98 [7.79, 8.17]	7.73 [7.57, 7.98]	0.054
IL18	9.02 [8.68, 9.35]	8.51 [8.44, 8.65]	0.105
IL8	6.50 [5.93, 6.70]	6.84 [6.39, 6.96]	0.165
PDGF-B	10.69 [10.66, 10.80]	10.83 [10.81, 10.91]	0.165
IL6	3.77 [3.52, 4.51]	4.26 [3.96, 4.76]	0.190
CCL2	10.71 [10.50, 10.97]	10.91 [10.84, 11.16]	0.190
CRTAM	5.21 [5.07, 5.37]	5.04 [4.89, 5.14]	0.190
VEGF-C	2.99 [2.40, 3.20]	3.15 [2.95, 3.65]	0.190
CCL8	7.58 [7.37, 8.14]	8.08 [7.83, 8.46]	0.190
CCL3	5.95 [5.77, 6.47]	5.79 [5.49, 5.98]	0.217
PDL2	2.74 [2.54, 2.88]	2.52 [2.27, 2.61]	0.217
EGF	10.47 [9.84, 10.95]	9.78 [9.40, 10.33]	0.280
IL7	5.82 [5.00, 6.15]	6.16 [5.45, 6.41]	0.355
TNFRSF9	5.84 [5.73, 5.93]	5.72 [5.51, 5.82]	0.396
PIGF	8.47 [8.36, 8.50]	8.57 [8.42, 8.75]	0.396
CXCL11	6.45 [6.22, 7.19]	6.97 [6.37, 7.62]	0.396
GZMH	4.99 [4.62, 5.02]	4.64 [4.28, 5.20]	0.396
CCL4	8.15 [7.55, 8.87]	7.92 [7.54, 8.07]	0.396
CX3CL1	6.69 [6.30, 7.11]	6.79 [6.63, 7.30]	0.396
VEGFA	8.65 [8.37, 9.14]	8.87 [8.66, 9.06]	0.396
CD40L	8.06 [7.08, 8.25]	7.76 [6.46, 8.07]	0.440
CD244	6.30 [5.88, 6.49]	6.07 [5.90, 6.26]	0.440
HO1	12.28 [12.01, 12.44]	12.12 [11.87, 12.40]	0.440
CXCL10	7.97 [7.24, 8.80]	8.36 [7.86, 9.04]	0.440
MMP12	6.85 [6.58, 7.48]	7.25 [6.84, 7.48]	0.440
IL10	2.53 [2.33, 2.70]	2.43 [2.27, 2.54]	0.447
TRAIL	8.18 [7.67, 8.26]	8.19 [8.02, 8.33]	0.487
ICOSLG	6.61 [5.73, 6.76]	5.80 [5.63, 6.92]	0.487
CSF1	7.56 [7.42, 7.67]	7.66 [7.50, 7.76]	0.487
GZMA	5.26 [5.21, 5.50]	5.23 [5.11, 5.48]	0.537
LAMP3	4.93 [4.33, 5.26]	4.98 [4.56, 5.49]	0.537
CCL20	5.87 [5.38, 6.17]	5.64 [5.21, 5.94]	0.537
FGF2	0.94 [0.76, 1.28]	0.86 [0.66, 1.03]	0.556
CD4	0.60 [0.43, 0.69]	0.60 [0.46, 0.88]	0.589
TWEAK	8.74 [8.48, 9.00]	8.88 [8.71, 8.97]	0.589
RSF4	3.26 [2.86, 3.53]	3.29 [3.16, 3.49]	0.589
CXCL13	8.88 [8.61, 8.99]	8.89 [8.73, 9.15]	0.589
IL12RB1	1.92 [1.75, 2.06]	1.78 [1.68, 1.98]	0.589
CD8A	9.01 [8.64, 9.45]	9.54 [8.81, 9.78]	0.643
CASP8	6.95 [6.38, 7.46]	6.82 [6.07, 7.30]	0.643
IL4	1.30 [1.30, 1.30]	1.51 [1.15, 1.52]	0.655
CXCL9	7.64 [6.94, 8.40]	7.65 [7.07, 8.58]	0.700
GAL1	6.26 [6.06, 6.33]	6.27 [6.11, 6.43]	0.700
MMP7	11.32 [11.14, 11.69]	11.38 [11.22, 11.63]	0.700
ANG2	4.77 [4.59, 4.91]	4.76 [4.57, 5.11]	0.700
NOS3	1.42 [1.05, 1.54]	1.43 [1.12, 1.45]	0.711
CA9	3.92 [3.66, 4.42]	3.94 [3.52, 4.20]	0.758
ADA	2.44 [2.02, 2.67]	2.46 [2.27, 2.68]	0.758
PDCD1	3.18 [2.91, 3.80]	3.35 [3.10, 3.50]	0.758
KLRD1	5.67 [5.47, 5.95]	5.76 [5.59, 5.99]	0.758
CCL7	2.72 [2.40, 3.31]	2.75 [2.64, 3.09]	0.817
GAL9	7.72 [7.25, 7.93]	7.58 [7.37, 7.73]	0.817
TGFB1	2.47 [2.24, 2.68]	2.47 [2.29, 2.86]	0.817
FasL	5.77 [5.67, 6.41]	6.03 [5.64, 6.22]	0.817
PDL1	4.86 [4.59, 5.16]	4.78 [4.58, 5.03]	0.817
CCL23	10.02 [9.81, 10.08]	9.92 [9.77, 10.18]	0.817
TNFRSF21	7.84 [7.64, 7.98]	7.84 [7.60, 7.93]	0.817
IL12	6.45 [5.93, 6.96]	6.42 [6.23, 6.57]	0.817
ARG1	3.33 [2.90, 4.21]	3.94 [2.74, 4.45]	0.866
PTN	2.23 [1.32, 3.35]	1.80 [1.20, 2.81]	0.866
CD40	10.87 [10.65, 11.09]	10.80 [10.61, 11.13]	0.877
TNFSF14	5.01 [4.70, 5.46]	4.90 [4.60, 5.49]	0.877
CCL19	9.27 [8.31, 9.83]	9.10 [8.53, 9.59]	0.877
HGF	8.23 [8.03, 8.30]	8.16 [8.04, 8.45]	0.877
CD70	4.48 [4.33, 4.63]	4.48 [4.21, 4.71]	0.877
GZMB	3.71 [3.50, 3.79]	3.72 [3.15, 4.22]	0.877
CD83	2.46 [2.22, 2.92]	2.55 [2.46, 2.62]	0.877
ADGRG1	2.03 [1.79, 2.61]	2.28 [1.72, 3.20]	0.934
ANG1	10.30 [10.08, 10.41]	10.27 [10.19, 10.35]	0.939
VEGRF2	7.39 [7.24, 7.59]	7.40 [7.26, 7.55]	0.939
CD27	7.84 [7.52, 8.28]	7.99 [7.95, 8.20]	0.939
TNFRSF12A	6.32 [6.14, 6.44]	6.34 [6.02, 6.56]	0.939
CD5	4.70 [4.47, 5.03]	4.75 [4.55, 4.85]	0.939
NCR1	4.25 [4.04, 4.62]	4.29 [4.11, 4.48]	0.939

DCN	4.95 [4.87, 4.98]	4.93 [4.83, 5.28]	0.939
SDF1	1.20 [1.10, 1.34]	1.25 [1.09, 1.29]	0.939
CD28	1.49 [1.37, 1.87]	1.57 [1.46, 1.61]	1.000
MICAB	4.10 [3.94, 4.25]	4.14 [3.86, 4.37]	1.000

IQR= Inter quartile range
